# Necrotizing Fasciitis of the Extremities: Microbial Spectrum, Antimicrobial Resistance, and Clinical Outcomes in a 7-Year Retrospective Cohort Study

**DOI:** 10.3390/medicina62091744

**Published:** 2026-09-10

**Authors:** Cosmin Vasile Obleaga, Polliana Mihaela Leru, Sergiu Marian Cazacu, Alexandru Marin Pascu, Livia Dragonu, Andreea Doriana Stanculescu, Dragos George Popa, Florin-Liviu Gherghina, Dragos Marian Popescu, Ion Vasile, Lidia Boldeanu

**Affiliations:** 1 General Surgery Department, Clinical Emergency Hospital Craiova, University of Medicine and Pharmacy Craiova, 200349 Craiova, Romania; cosmin.obleaga@umfcv.ro (C.V.O.); pascu.alexandru07@gmail.com (A.M.P.); 2 Internal Medicine Department, Colentina Clinical Hospital Bucharest, University of Medicine and Pharmacy Carol Davila, 020021 Bucharest, Romania; 3 Gastroenterology Department, Clinical Emergency Hospital Craiova, University of Medicine and Pharmacy Craiova, 200349 Craiova, Romania; 4 Infectious Disease Department, Pneumology and Infectious Disease Hospital Craiova, University of Medicine and Pharmacy Craiova, 200349 Craiova, Romania; livia.dragonu@umfcv.ro; 5 Intensive Care Department, Clinical Emergency Hospital Craiova, University of Medicine and Pharmacy Craiova, 200349 Craiova, Romania; andreea.stanculescu@umfcv.ro; 6 Plastic Surgery Department, Clinical Emergency Hospital Craiova, University of Medicine and Pharmacy Craiova, 200349 Craiova, Romania; dragos.popa@umfcv.ro; 7 Department of Physical Medicine and Rehabilitation, University of Medicine and Pharmacy of Craiova, 200349 Craiova, Romania; florin.gherghina@umfcv.ro; 8 Extreme Conditions Medicine Department, University of Medicine and Pharmacy Craiova, 200349 Craiova, Romania; dragos.popescu@umfcv.ro; 9 Dolj Section, Medical Sciences Academy Romania, 200349 Craiova, Romania; vasileion52@yahoo.com; 10 Department of Microbiology, Faculty of Medicine, University of Medicine and Pharmacy of Craiova, 200349 Craiova, Romania; lidia.boldeanu@umfcv.ro

**Keywords:** necrotizing fasciitis, lower limbs, upper limbs, plastic and reconstructive surgery, antibiotic resistance

## Abstract

*Background and Objectives*: Necrotizing fasciitis (NF) is characterized by rapid and aggressive infection of the subcutaneous tissue and fascia, and a high risk of death in case of delay in diagnosis and treatment. *Materials and Methods*: This 7-year retrospective study (May 2017–May 2024) evaluated 38 patients with upper- and lower-limb NF admitted to the surgery clinic at the County Emergency Hospital in Craiova, Romania. *Results*: 21.8% of the confirmed NF cases were monomicrobial; Gram-positive strains were predominant, but Gram-negative strains were also involved. The mortality rate was 28.9%; acute kidney injury appears as the only independent risk factor associated with mortality, although in univariate analysis, septic shock and high NLR and INR values were also factors associated with mortality. An increased resistance rate to antibiotics was recorded, with a 32.7% MDR rate; moderate to high resistance to piperacillin + tazobactam, most cephalosporins, most carbapenems (for Gram-negative strains), and quinolones was noted. *Conclusions*: Multidisciplinary management, comprising intensive medical support, prompt targeted antibiotic therapy, and immediate surgical intervention, was critical for achieving favorable patient outcomes. Increasing antibiotic resistance may alter the prognosis in patients with NF.

## 1. Introduction

Necrotizing fasciitis (NF) is a progressive, necrotizing infection of the fascia and subcutaneous adipose tissue; the destruction is confined to the skin and underlying tissue, but does not involve the muscle [1,2]. Microbiologically, this pathology is divided into type 1 (polymicrobial) and type 2 (monomicrobial) [3]. Type 1 of non-causative fasciitis occurs more frequently on the trunk and perineum in immunocompromised patients (diabetes mellitus, chronic renal failure, neoplasia under oncological treatment) and is due to aerobic and anaerobic saprophytic microorganisms near the site of infection. In contrast, type 2 is less frequent and usually occurs on the limbs, affecting apparently healthy patients, sometimes without associated comorbidities, or may occur as a complication of surgical wounds or older injury [4,5,6,7,8]. Clinical presentation can overlap, and some necrotizing fasciitis of the limb can also be polymicrobial. Type 3 NF (associated with Vibrio vulnificus) and type 4 (fungal NF in immunosuppressed patients) were also described [8,9]. It is considered that Streptococcus pyogenes (group A) and Staphylococcus aureus strains are the common pathogens associated with type 2, and toxic shock syndrome may also occur in the clinical course of this type [10,11,12,13,14,15,16,17], although Gram-negative strains such as E. coli necrotic toxin-secreting strain were also rarely described [18,19]; some studies showed a higher proportion of Gram-negative bacteria, reflecting a particular proportion of type 1 NF [8,14,20] or high proportion of Vibrio vulnificus NF [21]. A shift to monomicrobial and type 2 NF was noted in some studies [14,22]. These bacterial species spread rapidly through the fascia and release a variety of toxins, resulting in inflammatory changes in the microvascular walls that facilitate microvascular thrombosis [1,23]. This limits antibiotic penetration, while pyrogenic exotoxins drive rapid T-cell proliferation. Consequently, this triggers the cytokine production that precedes shock and multiorgan failure [24].

The early clinical diagnosis of patients with NF is often difficult to establish [25], thus leading to increased mortality. Local pain, swelling, and erythema are the most common signs (rarely simultaneous, although all appear in the course of the disease) [26], and the dynamic appearance of skin crepitations and necrosis guides the clinical diagnosis. Some studies have shown a more aggressive clinical course in Gram-negative NF [12]. Increased leukocyte and C-reactive protein levels, fluid and electrolyte imbalances, and the diagnosis of an infraclinical disease (diabetes mellitus, renal failure) are frequently encountered. The association of toxic shock and multiple organ failure at the time of diagnosis is a negative prognostic factor [25]. LRINEC score (Laboratory Risk Indicator for Necrotizing Fasciitis) incorporates white blood cell count, C-reactive protein, hemoglobin, sodium, creatinine, and glucose levels; it can predict both risk of disease presence and mortality. Values below 6 were considered to be at low risk of necrotizing fasciitis, but cannot completely rule out the presence of the disease [8,27]. Local skin changes consist of tenderness, crepitus, skin necrosis, and hemorrhagic blisters. The presence of crepitus suggests infection with anaerobic bacteria, which is useful for the treatment strategy. Imaging examinations include plain radiography (for gas gangrene), CT scan (extent of tissue infection, fascial swelling, inflammation, and gas formation), MRI scan (very rarely used in emergencies), and ultrasound (which is indicated in cases of gas gangrene) [25,28].

Managing patients with NF requires intensive care support, empirical broad-spectrum antibiotics, and prompt, often repeated surgical interventions. These surgeries involve collecting bacteriological samples, excisional debridements, fasciotomies, release incisions, and potentially amputations. Surgical intervention is crucial because localized ischemia can restrict the delivery and effectiveness of systemic antibiotic therapy [26]. For surviving patients, closing skin defects and restoring muscle strength pose significant post-operative challenges for plastic surgeons and physiotherapists. Addressing these issues is crucial for shortening hospital stays and facilitating social reintegration [29,30,31].

NF continues to carry a substantial mortality rate, with the majority of literature reporting figures ranging from 20% to 40% [12,32,33,34]. The etiological spectrum and antimicrobial susceptibility profiles may influence the prognosis and clinical outcomes of NF, with inadequate or suboptimal therapy in a subset of patients [21]. Escalating antibiotic resistance across various infectious diseases suggests that shifting etiologies could severely compromise patient survival and tissue recovery. Furthermore, regional variations in bacterial susceptibility complicate the establishment of universal antibiotic protocols. Consequently, local studies characterizing the etiological landscape, resistance patterns, and mortality in patients with NF are highly warranted.

## 2. Materials and Methods

We conducted a 7-year retrospective study involving 55 patients diagnosed and treated for necrotizing fasciitis at the County Emergency Hospital in Craiova between 1 May 2017, and 1 May 2024. Patient care was managed collaboratively by the General Surgery Clinic, the Intensive Care Unit (ICU), and the Plastic and Reconstructive Surgery Department. Cases were identified using DRG code M74.58 within the Hippocrate Hospital electronic database, with data verification using the available medical records. Analyzed variables included demographic characteristics, medical history, diagnostic methods, pharmacological and surgical interventions, survival rates, wound closure techniques, and social reintegration. The main objective was to assess the etiological pattern and antibiotic resistance in patients with NF. Secondary outcomes were in-hospital mortality rates, risk factors associated with in-hospital mortality, and amputation rates.

The study was approved by the Ethics Committee of the Craiova County Emergency Clinical Hospital (47254/23.10.2024) on the following bases: (1) The data were collected in a retrospective, observational, descriptive, and non-experimental study; (2) the study did not interfere with current medical care; (3) no substances were administered to the patients, and no biological samples were collected in the study; and (4) the data were collected and analyzed anonymously so that the confidentiality of patient data was not violated.

The inclusion criteria were as follows: all patients diagnosed with necrotizing fasciitis of the lower or upper limbs with complete data regarding medical history, the date of the initial medical consultation, the date and time of admission to a ward (surgery, ICU, or infectious diseases), pharmacological therapy, the date and time of surgical interventions, initial antibiotic therapy, and clinical follow-up. Data regarding comorbidities, laboratory values (hemogram, C-reactive protein, albumin, INR, urea, and creatinine levels), and the presence of acute kidney injury, septic shock, and LRINEC score (if available) were also recorded. Patients with incomplete data, as well as those transferred or discharged before surgery at the family’s request, were excluded. The diagnosis of necrotizing fasciitis of the lower limb was established based on clinical examination (necrotic fascial tissue, a lack of resistance of normally adherent muscular fascia to blunt dissection, the lack of bleeding of fascia during dissection, and the presence of foul smelling pus), imaging studies (X-ray or CT scan, when performed), intraoperative findings, antibiogram results, and the histopathological analysis of excised tissues.

Surgery with fasciotomy and debridement was performed under general anesthesia; deep samples of excised tissues were sent for both pathology and microbiological examination. Most patients undergo rapid surgical intervention associated with broad-spectrum antibiotics, fluid resuscitation, and vasopressors if hypotension occurs. Intensive care support was needed for almost all patients. Local therapy was performed twice daily using saline, hydrogen peroxide, and Betadine, with iterative debridement based on wound progression, completed by secondary suturing or split-thickness skin grafts. Cultures obtained in a sterilized surgical room or pus cultures collected from open wounds immediately after admission were analyzed. All samples were immediately inoculated into blood broth media, incubated for 18 h at 37 °C, and then inoculated on blood agar, chocolate agar, and Drigalski agar media, provided by BioMérieux (Salt Lake City, UT, USA); rapid transport in specialized media to protect strict anaerobes from oxygen exposure was employed. For anaerobic bacteria analysis, samples were inoculated onto specific media and incubated under anaerobic conditions using the Genbag system and an anaerobic generator. Following incubation, polymicrobial cultures were differentiated based on distinct macro-morphological colony characteristics, and each distinct morphotype was subcultured onto secondary media to achieve pure clonal isolation. Definitive identification and antimicrobial susceptibility testing of individual pure strains were performed according to EUCAST standards using the automated VITEK 2 system (BioMérieux), utilizing identification cards alongside AST-XN26, AST-N438, AST-GP67, AST-P592, and AST-ST03 susceptibility cards [35]. In parallel, standard phenotypic antibiotic testing was performed with a Kirby-Bauer disk diffusion test using antibiotic disks (Biomaxima S.A. (Cluj-Napoca, Romania). The cefoxitin disk diffusion test was used for MRSA detection; ESBL and carbapenemase production of Enterobacteriaceae strains was detected phenotypically [36] using the disk diffusion double disk method with kits provided by Rosco Diagnostica (Albertslund, Denmark). Briefly, a 0.5 McFarland inoculum derived from isolated pure cultures was deposited on a plate of Mueller–Hinton agar, the five Rosco tablets (meropenem alone and in three inhibitor combinations plus temocillin) were incubated overnight, and the zone-size differences were read according to manufacturer instructions to detect the presence of KPC, MBL, AmpC-porin, or OXA-48 carbapenemases.

**Figure 1:**
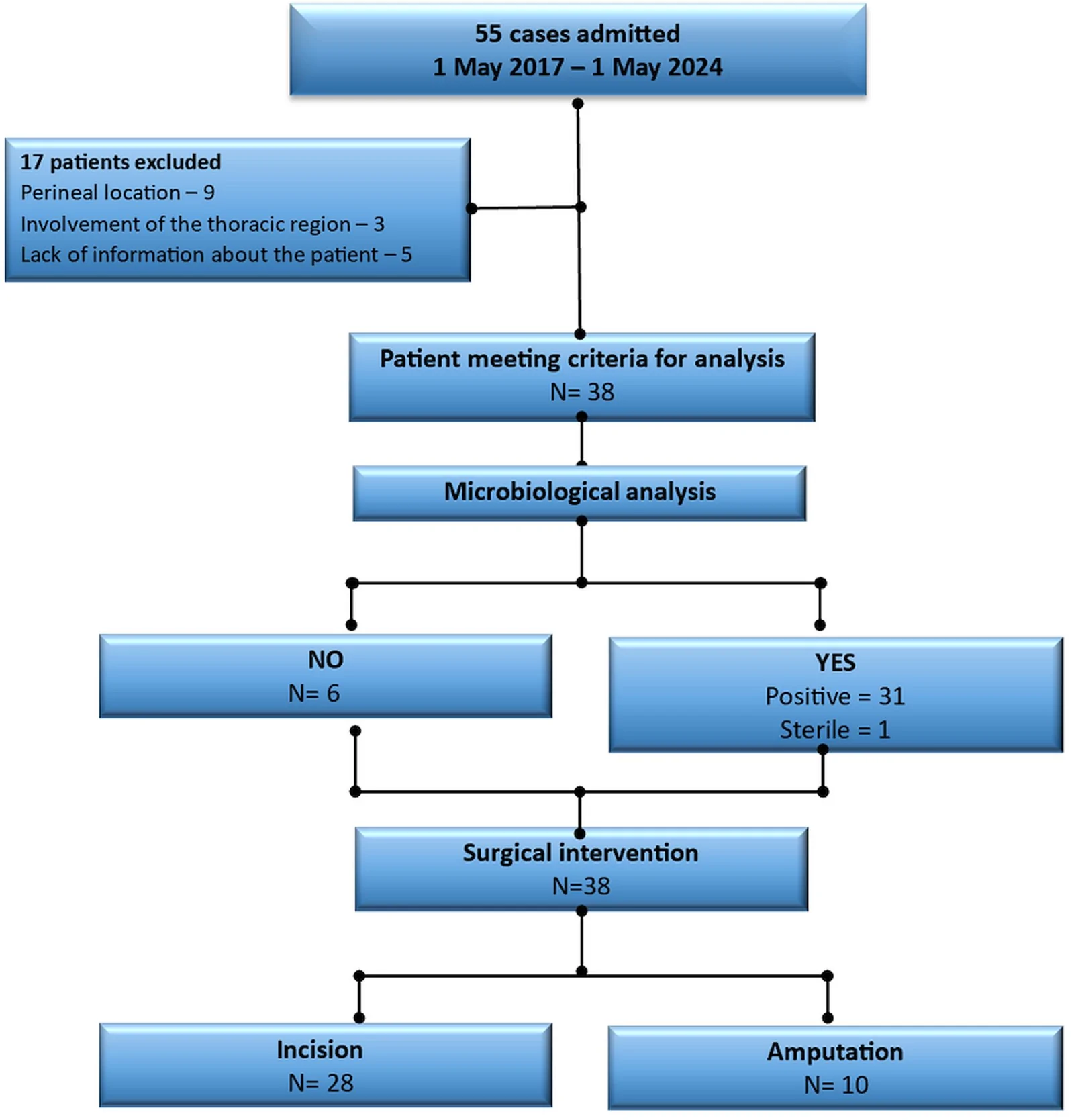
Details of patient flow selection in necrotizing fasciitis of the limbs.

Cultures were classified as monomicrobial or polymicrobial, Gram-positive, Gram-negative, or anaerobic, with the microorganism genus and species recorded. The proportions of cases with positive cultures of Staphylococcus (MRSA or MSSA), Streptococcus (including group A β-hemolytic strains), Enterococcus, Gram-negative strains, anaerobic flora, or fungi were noted. Isolated strains were classified as MDR according to the definition of the Clinical Laboratory Standards Institute (CLSI), the European Committee on Antimicrobial Susceptibility Testing (EUCAST), and the United States Food and Drug Administration (FDA), as acquired non-susceptibility to at least one agent in three or more antimicrobial categories [37]. All blood microbiological samples were also recorded (Figure 1).

Data were analyzed using Microsoft Excel with the XLSTAT 2016 add-on (Addinsoft SARL, Paris, France). Continuous variables were expressed as means with standard deviations and compared using the *t*-test or Mann–Whitney test depending on the normality of the distribution, assessed by the Shapiro–Wilk test. Categorical variables were presented as percentages and compared using chi-square tests or Fisher’s exact tests. Univariable and multivariable logistic regression analyses were performed to identify predictors associated with the primary endpoints (mortality and amputation). Due to the small sample size and the presence of complete or quasi-complete data separation among key predictors, standard maximum likelihood estimation was replaced by Firth’s Penalized Likelihood Logistic Regression to ensure stable and finite parameter estimates. Effect estimates are expressed as Odds Ratios (OR) derived from the penalized likelihood function. To maintain statistical integrity under sparse data constraints, 95% Confidence Intervals (CI) were computed using the Profile Likelihood method, and *p*-values were calculated using the Penalized Likelihood Ratio Test. A two-tailed *p*-value < 0.05 was considered statistically significant. All statistical analyses were conducted using R software (version 4.6.1).

## 3. Results

### 3.1. General Characteristics of Patients with NF

A total of 38 patients with necrotizing fasciitis of the limbs were included in this study, out of a total of 55 patients extracted with the disease-specific DRG code; 17 patients were excluded due to other locations (9 patients had perineal location, 3 patients had trunk location, 2 were discharged at family request, 1 patient was transferred, and 2 patients had incomplete data). The main characteristics of the 38 patients are presented in Table 1. It is important to note that CRP levels were available for only 13 patients at admission; therefore, the LRINEC score was not used in our study.

**Figure 2:**
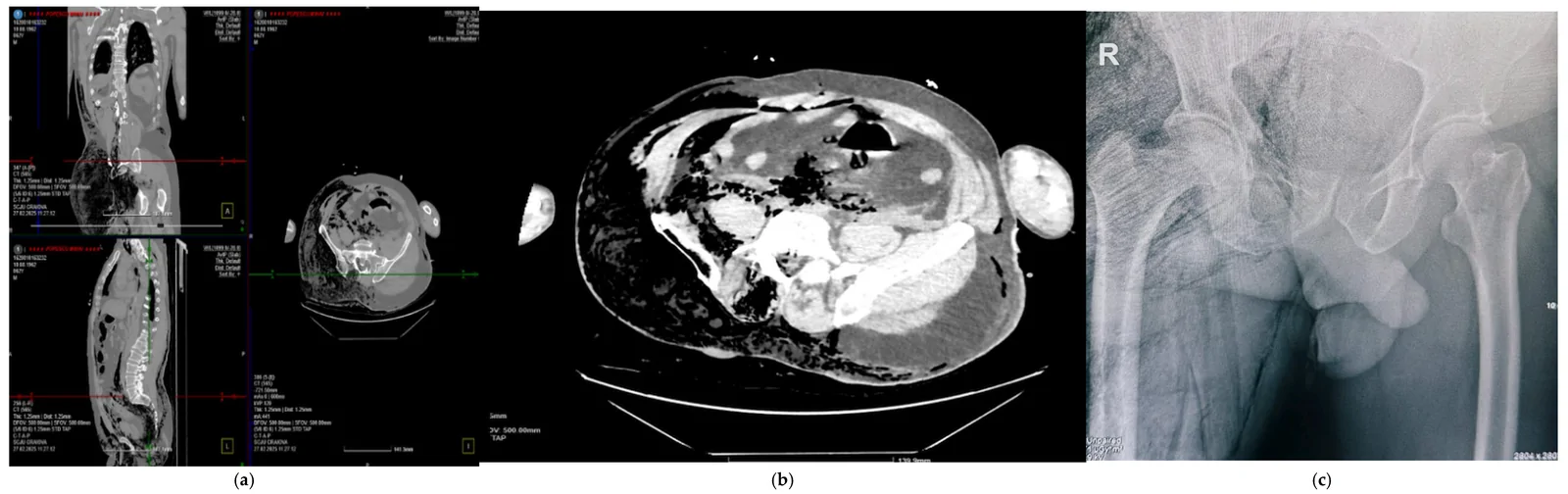
Imaging findings in a patient with fasciomyonecrosis: (**a**,**b**) computed tomography (CT) images demonstrating the extent of the disease; (**c**) plain radiograph (X-ray).

**Figure 3:**
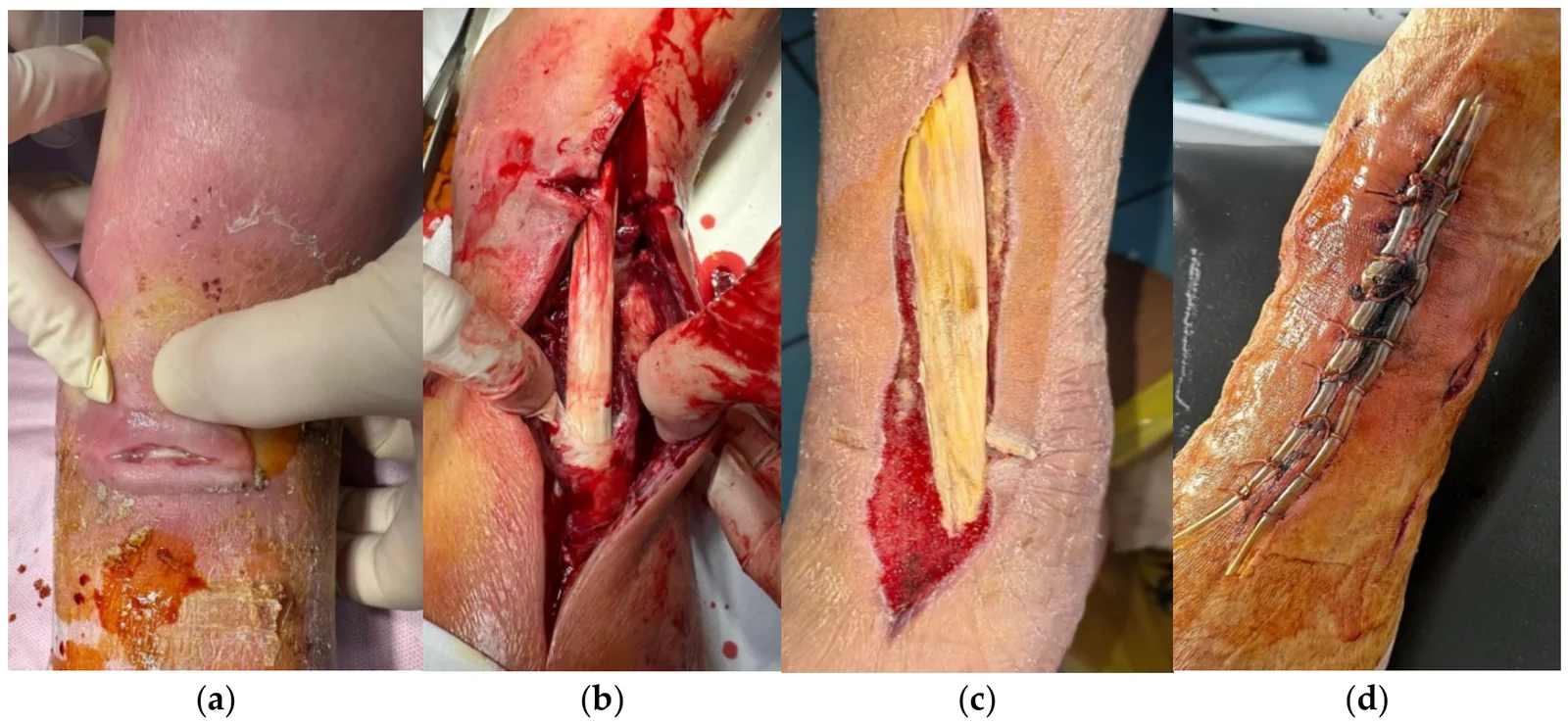
Necrotizing fasciitis of the lower leg in a patient with diabetes mellitus: (**a**) clinical aspect at the initial evaluation; (**b**) intraoperative findings; (**c**) wound appearance after negative pressure wound therapy (NPWT); (**d**) wound aspect following definitive closure of the soft tissue defect.

Most patients with necrotizing fasciitis were males (76.3%); the mean age was 57.5 years. Diabetes mellitus was the most frequent comorbidity (73.7%). Local pain was noted in almost all cases (94.74%); swelling and local heat were found in 89.5 and 44.7%, respectively. Cutaneous gangrene or obvious phlegmon was found only in 2 patients. Patient admission occurred 4 to 48 h after symptom onset. Imaging procedures (ultrasound, computed tomography) were used in only three patients with severe forms or uncertain diagnosis, raising suspicions of fasciitis or leading to a revised diagnosis of fasciomyonecrosis (Figure 2 and Figure 3). The final diagnosis was always intraoperative in all patients; in patients with initially limited lesions, the protocol was changed by increasing the scope of the intervention (multiple incisions vs. amputation).

In necrotizing fasciitis, surgical intervention served a dual purpose: controlling the infection and harvesting biological specimens—such as pus, affected fascia, and necrotic tissue—for bacteriological analysis, obtained after onset of empirical antibiotherapy, because guidelines recommend prophylactic antibiotherapy before surgery. Preoperative empiric antibiotics were initiated and later adjusted according to the antibiogram. Negative pressure wound therapy (NPWT) was utilized in 31.6% of the cohort (*n* = 12), effectively reducing hospital stays, dressing frequency, and antibiotic requirements. Furthermore, this therapy accelerated the timeline to definitive surgical closure by promoting rapid wound healing, which was characterized by healthy granulation tissue formation and the absence of residual pathological lesions.

### 3.2. Bacterial Flora in Necrotizing Fasciitis

Among the 38 patients with NF, culture and susceptibility results were unavailable in 6 cases, and one culture was sterile. In the remaining 31 patients, one or more pathogens were isolated, yielding a total of 49 bacterial strains. The most prevalent microorganism was *Staphylococcus* spp. (predominantly *S. aureus*) in 14 cases (45.2%), followed by *Streptococcus* spp. (*n* = 7, 22.6%). *Acinetobacter*, *Escherichia coli*, and *Klebsiella* spp. were each isolated in six cases (19.4%). *Enterococcus*, *Proteus*, *Pseudomonas*, and *Serratia* species were infrequently encountered, and a single case of anaerobic *Peptostreptococcus* fasciitis was recorded (Table 2).

Gram-negative bacterial strains were noted more frequently in polymicrobial fasciitis (9 were monomicrobial and 10 were polymicrobial).

Lower limb necrotizing fasciitis was identified in 32 cases, comprising 11 polymicrobial infections (34.4%) and 14 monomicrobial infections; in 6 cases, no culture was recorded, and in another case, the culture was sterile. Gram-positive bacteria predominated (over two-thirds of the cases), although Gram-negative pathogens were also isolated (more often *E. coli*, *Klebsiella*, and *Acinetobacter* spp.). Adequate therapy was recorded in half of the cases; inadequate therapy and not assessed susceptibility were noted in 25% each. The mortality rate was 28.1%.

Upper limb fasciitis was recorded in 6 cases, with one monomicrobial and 6 polymicrobial; four were determined by Gram-positive bacteria (2 *Staphylococcus aureus*, one *Streptococcus* spp., and one *Enterococcus*), and 3 were produced by Gram-negative strains (one *Acinetobacter* and two *Klebsiella* strains). 3 of 6 patients had diabetes (50%), but only one patient was obese. No vacuum was needed, but surgery was performed in all cases; no surgical reinterventions were required. The mortality rate was 33.3%. Antibiogram-guided therapy was appropriate in 66.7% of cases. In contrast, treatment was inadequate in one case, while susceptibility analysis was omitted in another.

**Figure 4:**
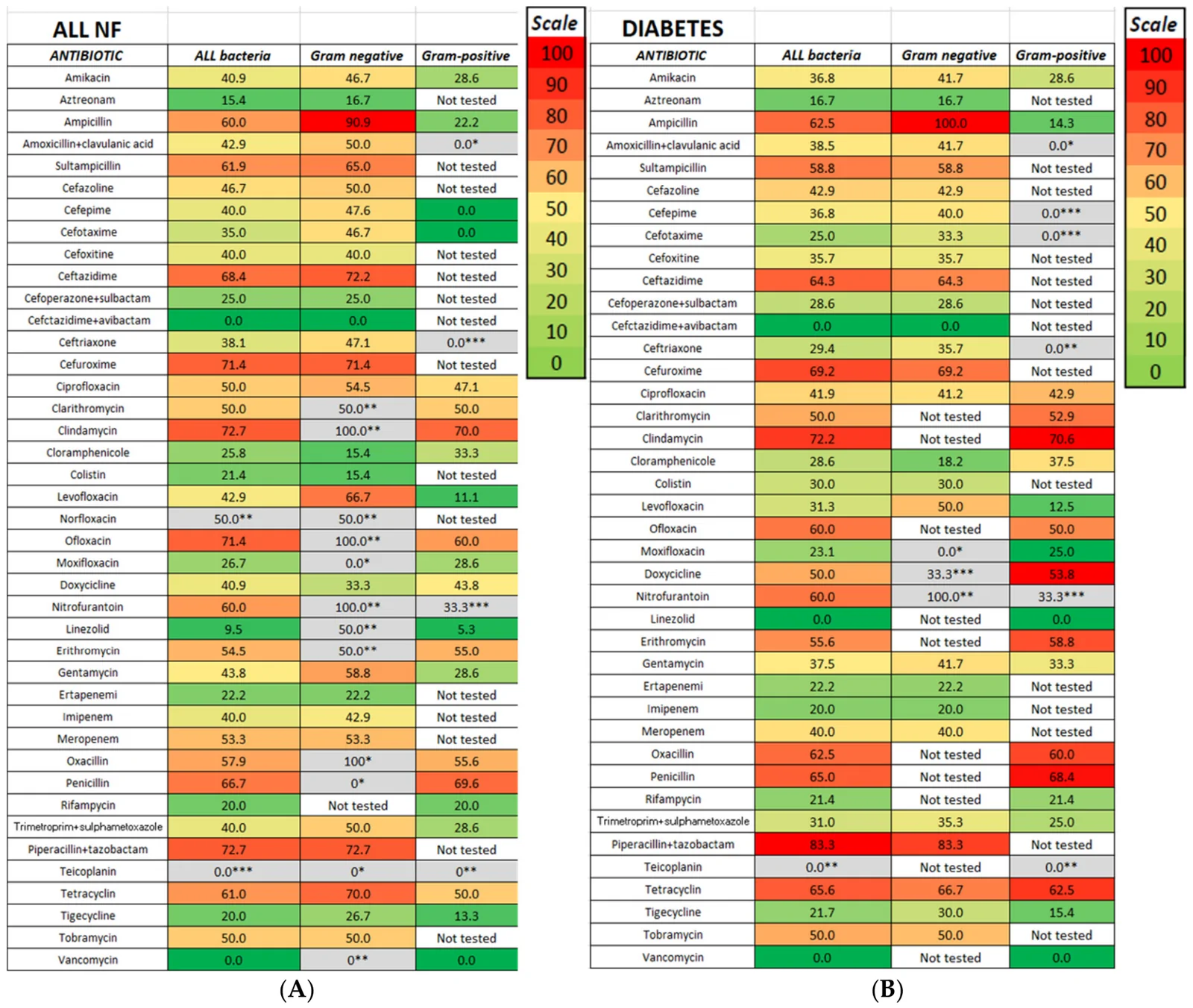
(**A**) Antibiotic resistance in all patients with NF. (**B**) Antibiotic resistance in diabetic patients with NF. * = only one strain tested, ** = only 2 strains tested, *** = only 3 strains tested.

The most commonly used combinations of antibiotics in our patient group were either a third-generation cephalosporin (mostly cefepime + sulbactam) or a carbapenem plus an aminoglycoside plus metronidazole for anaerobic cover; empirical antibiotherapy was adjusted (if needed) after antibiogram results were received and depending on clinical general and local evolution. In all patients with necrotizing fasciitis, low-level resistance in both Gram-positive and Gram-negative strains was observed only for tigecycline, with rates between 20 and 25%. For Gram-positive strains, cefepime, cefotaxime, levofloxacin, linezolid, and vancomycin were associated with resistance rates of 0–10%; the resistance rate to moxifloxacin was 28.6%, whereas moderate or high resistance rates were noted for ciprofloxacin, clindamycin, and erythromycin (Figure 4A). For Gram-negative strains, aztreonam, chloramphenicol, colistin, and combinations of ceftazidime + avibactam and cefoperazone + sulbactam have low resistance rates; high or moderate resistance was recorded for most cephalosporins, quinolones, and most carbapenems (except ertapenem). All *Acinetobacter*, 2 of 6 *Klebsiella* strains, and a *Serratia* strain were MDR; 2 ESBL *E. coli* strains and 5 MRSA *S. aureus* strains were also identified, but no vancomycin-resistant Enterococcus (VRE) strains were detected (total resistance rate reported for the 49 strains tested was 32.7%). Details regarding the antibiotic resistance for the three most frequent Gram-positive and Gram-negative strains are detailed in Supplementary Figures S1 and S2.

### 3.3. Bacterial Flora and Susceptibility in Patients with Diabetes

Among 28 diabetic patients, the bacteriology was nearly equally distributed between Gram-positive (20 cases) and Gram-negative (18 cases) pathogens, with *Staphylococcus aureus* and *Streptococcus* spp. being the most frequent isolates. Patients with diabetes and NF exhibited a higher frequency of *Staphylococcus* spp. infections than those without diabetes (42.9%, *n* = 12/28 vs. 20%). Susceptibility was similar to that of the whole patient group, with high resistance to most cephalosporins (among Gram-negative isolates), erythromycin, ciprofloxacin, and carbapenems (Figure 4B). Both mortality and amputation rates in diabetic patients were 32.1% (9 of 10 cases of amputations occurred in diabetics). Details regarding the antibiotic resistance in diabetic patients with NF for the three most frequent Gram-positive and Gram-negative strains are found in Supplementary Figures S3 and S4.

### 3.4. Antibiotic Resistance in Relation to the Upper or Lower Limb Location of Necrotizing Fasciitis

**Figure 5:**
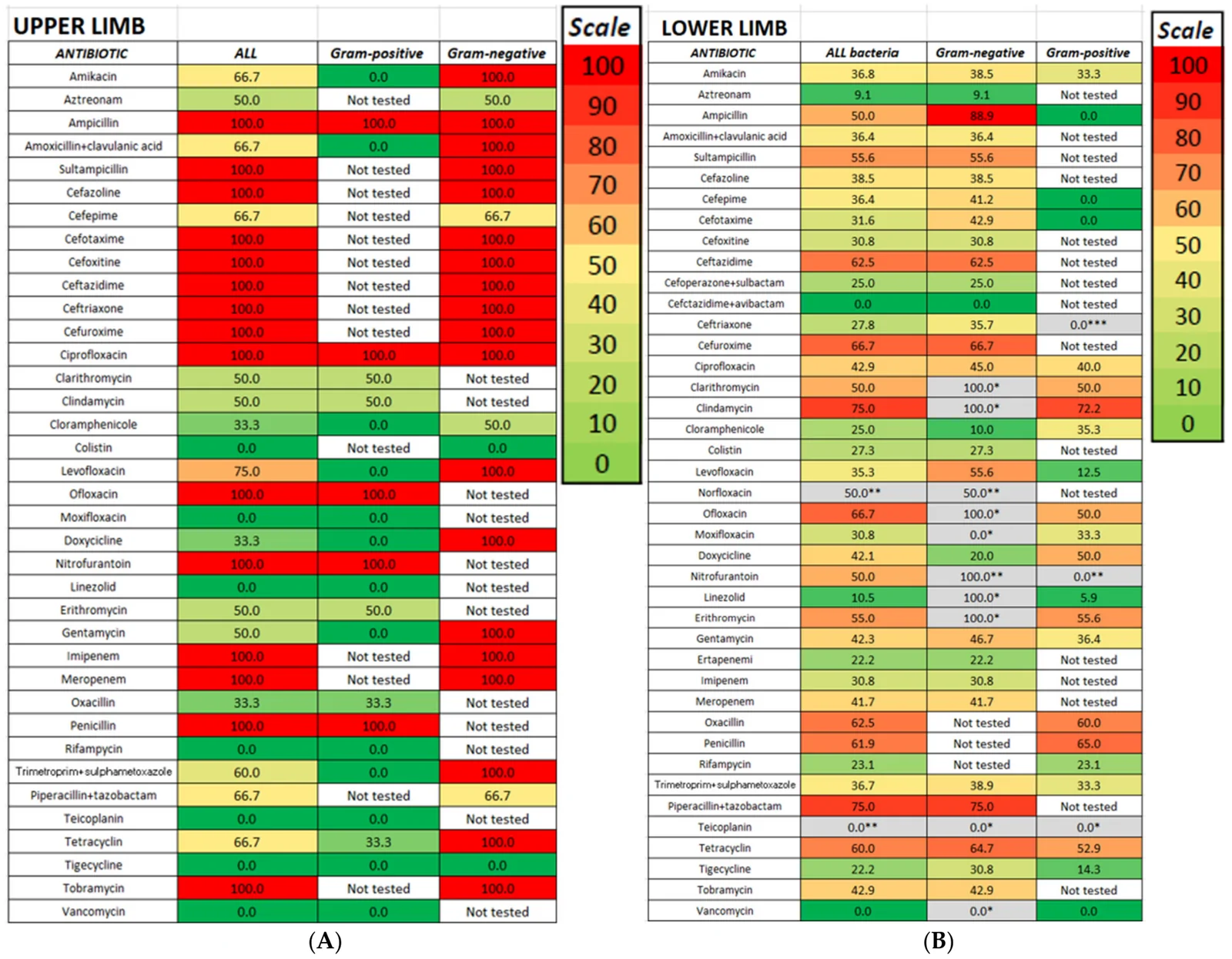
(**A**) Antibiotic resistance in patients with NF of the upper limb. (**B**) Antibiotic resistance in patients with NF of the lower limb. * = only one strain tested, ** = only 2 strains tested, *** = only 3 strains tested. For upper limb NF, most antibiotics were tested for 1–3 strains.

Susceptibility patterns depended on the anatomical location of NF. Lower extremity isolates demonstrated high in vitro susceptibility to tigecycline, ertapenem, ceftazidime-avibactam, and cefoperazone-sulbactam, with the latter two evaluated only against Gram-negative pathogens. In upper extremity infections, however, the highest susceptibility rates were observed for moxifloxacin, vancomycin, linezolid (Gram-positive panel), and colistin (Gram-negative panel) (Figure 5A,B).

### 3.5. Risk Factors for Mortality in Patients with Fasciitis

In patients with NF of the limbs, risk factors associated with mortality in univariate analysis were high urea and creatinine levels (in context of acute kidney failure), Gram-negative strains, septic shock, high INR and NLR levels, and low platelet count; high WBC and CRP, low Hb values, obesity, and peripheral vascular disease were more frequent in deceased patients, did not attain statistical significance, and CCI. Charlson comorbidity index also trended higher in deceased patients, without statistical significance (Table 3).

Due to the small sample size (*n* = 38) and the presence of complete or quasi-complete data separation regarding AKI, standard Maximum Likelihood Estimation would break down, yielding divergent parameter estimates and unstable standard errors. To resolve this boundary issue, Firth’s penalized likelihood logistic regression was applied, which penalizes the log-likelihood function using the Jeffries invariant prior, successfully eliminating first-order asymptotic bias and guaranteeing finite, reliable coefficient estimates. We selected demographic and clinical parameters associated with a *p*-value < 0.10 in univariate analysis; urea and creatinine were excluded because of the high correlation with AKI presence. The remaining 8 parameters (obesity, Gram-negative versus Gram-positive flora, AKI, septic shock, Hb level, platelet count, NLR, and INR) were evaluated by using multicollinearity analysis, confirming that all covariates fell well within safe, operational boundary limits (all VIFs < 2.15, mean VIF = 1.45).

Firth penalized Likelihood logistic regression was then performed. On crude univariate evaluation, AKI (crude OR = 67.13; 95% CI: 7.07–1851.52, *p* < 0.0001), Septic shock (crude OR = 6.82; 95% CI: 1.46–40.54), *p* = 0.013), INR > 1.5 (crude OR = 13.91, 95% CI: 1.81–335.71), and NLR > 10 (crude OR = 13.31; 95% CI: 1.73–319.45, *p* = 0.0005) were significantly correlated with increased mortality. The model explained a substantial proportion of the outcome variance, with a McFadden Pseudo-R^2^ of 0.5721 and a Nagelkerke Pseudo-R^2^ of 0.6985. On multivariate analysis, the presence of AKI emerged as the strongest factor associated with mortality (adjusted OR = 24.31, 95% CI: 1.21–1554.20, *p* = 0.019), whereas septic shock, Gram-negative infection, elevated NLR, and high INR have lost their independent statistical significance and did not remain independently associated with mortality after adjustment for AKI. Except for AKI, none of the other parameters have reached statistical significance in multivariate analysis (*p* > 0.05), possibly due to the small sample size (Table 4).

Multivariable analysis in small samples may be associated with a low number of events per variable (EPV). Our initial model of 11 deaths and 8 variables gives us an unacceptable low EPV of 1.375. Although formal multicollinearity analysis did not reveal any interaction between our variables (all VIFs < 2.15), physiological markers (NLR, INR, PLT) are deeply intertwined downstream indicators of severe systemic sepsis and subsequent multi-organ failure. Including all five significant univariable predictors simultaneously would break the model’s structural stability and inflate the parameter burden. Therefore, to aggressively combat overfitting and respect biological redundancy, after univariate analysis, in the multivariable consolidation step, the final multivariable framework was restricted strictly to the two primary risk factors: acute kidney injury and septic shock. Transitioning to our final parsimonious model successfully optimized the EPV to a mathematically stable 5.5 (11 deaths/2 variables). In this final adjusted model, AKI remained a robust independent predictor of mortality.

### 3.6. Risk Factors Associated with Amputation in Patients with Necrotizing Fasciitis of the Limb

In patients with NF of the extremities, no risk factors for amputation were recorded outside the local clinical evolution; all analyzed parameters had no statistical significance, with *p*-value > 0.05 (Table 5). No multivariate analysis was therefore useful.

## 4. Discussion

Necrotizing fasciitis (NF) is a fulminant, rapidly progressive, and potentially life-threatening infectious disease recognized in the literature as a critical surgical emergency. The outcomes in our 38-patient cohort align with published data from similar studies, underscoring the decisive impact of prompt intervention, effective comorbidity management, and a multidisciplinary approach.

The clinical and demographic characteristics of our study cohort align with established risk factors for NF. The cohort’s mean age was 57.5 years, with a distinct male predominance, consistent with previously published data [8,16,38]. The distribution of comorbidities reveals a high prevalence of diabetes mellitus (28 patients; 73.7%), a condition recognized as a determinant of negative prognosis in NF by affecting microcirculation and altering the immune response. Obliterative arteriopathy and obesity affected 23.7% and 26.3% of the cohort, respectively, with both factors recognized as indicators of a worse clinical course. 10 patients (26.3%) presented with acute kidney failure, which represents a significantly higher percentage than that reported in some cohorts and underlines the degree of vulnerability of the studied group.

Computed tomography (CT) was performed selectively, revealing classic signs of necrotizing fasciitis (NF). These key findings included gas within the fascial planes, soft tissue destruction, and the spread of infection into neighboring regions, such as the perineum, scrotum, and testes. Although such images can guide the diagnosis, international guidelines emphasize that imaging investigations should not postpone surgical intervention when clinical suspicion is strong.

Microbiological analysis revealed the presence of several pathogens. The most frequent results were *Staphylococcus* spp. (identified in multiple samples), *Streptococcus* spp., *Enterococcus* spp., and *Enterobacteriaceae*. This distribution confirms the predominance of Gram-positive bacteria, especially *Staphylococcus* and *Streptococcus*, typical of type II necrotizing fasciitis, but also highlights the possible polymicrobial nature in some cases; Gram-negative strains were, however, encountered in close to 50% of the cases, almost half being polymicrobial.

In our study, antibiotic resistance was significant, with no single antibiotic demonstrating susceptibility in more than 80% of both Gram-positive and Gram-negative bacterial strains; aztreonam, tigecycline, colistin, chloramphenicol, cefoperazone + sulbactam, ceftazidime + avibactam, and ertapenem are associated with the lowest resistance rate in Gram-negative infection, whereas linezolid, cefepime, ceftriaxone, levofloxacin, tigecycline, and vancomycin showed the highest susceptibility in Gram-positive cases. A combination of two antibiotics covering both the Gram-positive and Gram-negative spectrum was needed in our patient group. A third antibiotic for anaerobic flora is usually recommended, but in our patients, only one had an anaerobic strain (*Peptostreptococcus*). Resistance to carbapenems was significant, except for ertapenem (28.6% resistance), contrary to other studies showing over 90% sensitivity to carbapenems [8,14]. The resistance to levofloxacin was low for Gram-positive (11.1%) strains but high in Gram-negative NF. The resistance to piperacillin + tazobactam was higher (over 66%) in Gram-negative NF, contrary to other studies [8,14]. Elevated rates of in vitro resistance to clindamycin have been observed in our study; multiple authors advocate for an empiric regimen combining clindamycin with a beta-lactam [8,12,39]. The Guidelines of the American Society of Infectious Diseases recommend the combination of ampicillin-sulbactam plus ciprofloxacin plus clindamycin [39]. In our study, in vitro susceptibility suggests that combining a beta-lactam, moxifloxacin, levofloxacin, or vancomycin with aztreonam may be useful for patients with necrotizing fasciitis. However, given the small sample size, these findings and therapeutic combinations must be cautiously considered, since in vitro susceptibility is not always concordant with the clinical efficacy of antibiotic therapy.

NF occurs less frequently in the upper limbs than in the lower extremities, though both locations share a similar microbiological profile dominated by *Streptococcus* and *Staphylococcus* species; local trauma often serves as a key predisposing factor [39]. Furthermore, higher mortality rates are strongly linked to renal and hepatic impairment, thrombocytopenia, or the presence of necrotic tissue extending proximally to the elbow at the time of admission [39].

Diabetes mellitus represents one of the most frequent comorbidities and risk factors for NF [38,40], with a 44.5–72.3% prevalence reported [40]. The presence of diabetes mellitus was associated with a high risk of amputation (OR 12.32) [14]. In this study, diabetes was prevalent in 81.8% of cases, with Gram-positive and Gram-negative bacteria occurring in nearly equal proportions, though *Staphylococcus* infections showed a notably higher incidence; 8 cases were polymicrobial (28.6%). High susceptibility to infection, altered skin wound healing, micro- and macrovascular complications, and local neuropathy with subsequent delay in proper care of the wounds may explain the predisposition for a lengthier local evolution and increased risk for amputation, especially in cases associated with diabetic foot lesions [40]. No particular antibiotic resistance in diabetics compared to non-diabetics was recorded.

Surgical treatment played a central role; interventions included successive debridements, fasciotomies, and, in cases with extensive involvement, amputation. The amputation rate in our cohort was 26.3%, almost exclusively noted in patients with diabetes mellitus, and aligns with published literature, which reports values between 15% and 50% depending on disease severity and time to presentation. Early amputation has been advocated in patients with hemorrhagic bullae, high LRINEC scores, peripheral vascular disease, and bacteremia [41]. Published studies have associated amputation with several risk factors such as age > 60, male sex, diabetes, chronic wound as etiology, high LRINEC score, multiple injury sites, delayed presentation, high LDH on admission, low RBC counts, low albumin and sodium < 130 mEq/L [8,14,33]; in diabetic patients, a high Wagner score and low albumin level predicted amputation [42]. Usually, amputation is indicated in cases with irreversible necrosis associated with sepsis and failed multiple debridements [8,43]. Reintervention was necessary in 28.9% of patients, reflecting the progressive and sometimes recalcitrant nature of the infection. The need for a therapeutic vacuum was present in 50% of patients, confirming the usefulness of this method in controlling secretions, reducing edema, and preparing for reconstruction. Plastic surgery had an important impact on the reconstructive phase, facilitating wound healing through dermo-epidermal grafts or secondary closures. Although the literature recommends early involvement of the plastic surgeon, in practice, his intervention is often postponed for logistical reasons, a situation also reflected in our group.

The observed mortality rate in our cohort (28.9%, *n* = 11) aligns with findings from international literature, where reported rates range between 15% and 40% [12,17,22,32,33]. Analysis of fatal cases highlighted the presence of unfavorable prognostic factors: late onset, multiple comorbidities (especially diabetes and renal failure), advanced age, the onset of septic shock or MODS, and several biological parameters, such as high WBC count, high creatinine values, high INR, and low platelet counts [8,13,33]. In our patient group, most deceased patients presented clinical criteria for septic shock since admission and developed acute kidney injury. In addition, critically ill patients with necrotizing fasciitis, particularly those with septic shock and multiple organ dysfunction syndrome, are at increased risk of stress-related upper gastrointestinal bleeding, emphasizing the importance of gastrointestinal prophylaxis and early recognition of digestive complications during intensive care management [44]. In univariate analysis, the presence of acute kidney injury, septic shock, high INR, and high NLR values were associated with high mortality risk; however, in multivariate analysis, only AKI presence was demonstrated as an independent prognostic factor. The possible explanation was related to the fact that most of the other factors (sepsis, INR, NLR, but also Gram-negative infection or platelet count) may be closely correlated to the appearance of AKI.

Overall, the results obtained confirm the importance of early diagnosis, aggressive surgical intervention, and an integrated multidisciplinary approach, including intensive care, appropriate antibiotic therapy, reconstructive plastic surgery, and complete microbiological evaluation.

We acknowledged several study limitations. The unicentric character of the study may limit the generalizability of the obtained data; a future multicentric study in our region may overcome this limitation. The study group was small, which may impact the statistical power of the findings; however, in most monocentric studies regarding necrotizing fasciitis, the number of patients was similar. Some patients had no isolated bacteria or antibiotic susceptibility. The lack of data (especially for CRP values at admission) did not permit us to evaluate the importance of the LRINEC score for mortality prediction. We also acknowledged some heterogeneity in antibiotic susceptibility testing during the study period; a 7-year timeline is long enough to witness massive shifts in global and localized bacterial evolution, Clinical and Laboratory Standards Institute (CLSI) or EUCAST guidelines frequently update their Minimum Inhibitory Concentration (MIC) criteria, and therefore a bacterial strain labeled “susceptible” in the first year of the study might be flagged as “resistant” by Year 7 purely due to changing lab definitions, masking or exaggerating the true rate of genetic adaptation. Potential transitions in hospital laboratory infrastructure, such as moving from manual disk diffusion to automated susceptibility platforms, or using different cards, may introduce technological heterogeneity; however, in our hospital, VITEK-2 was available over all 7 years of our study. The pandemic period may have significantly impacted patient care and mortality. Significant differences between the in vitro susceptibility and clinical efficacy may be encountered in patients with fasciitis, and local ischemia may impede the local activity against causal bacteria. Future multicentric or prospective studies may overcome some of the above-mentioned limitations.

## 5. Conclusions

The study supports the need to develop standardized protocols for suspected necrotizing fasciitis, as well as to train medical personnel in recognizing early symptoms to reduce mortality and severe complications associated with this condition. Rising antibiotic resistance, compounded by an increasingly heterogeneous bacterial etiology, threatens to elevate mortality rates and complicate therapeutic management. Consequently, optimizing the timing of surgical intervention and fostering interdisciplinary collaboration represent pivotal strategies for mitigating mortality and preventing severe, life-threatening complications.

## Figures and Tables

**Table 1:** General characteristics of patients with necrotizing fasciitis.

Age mean ± standard deviation (min–max)	57.5 ± 13 (20–90)
Gender M/F (%M)	29/9 (76.3)
Site (%)	
-Lower limb	32 (84.2)
-Upper limb	6 (15.8)
Symptoms (%)	
-local pain	36 (94.7)
-swelling	34 (89.5)
-local warmth	17 (44.7)
-cutaneous necrosis	10 (26.3)
-systemic deterioration	25 (65.8)
Onset to admission interval (%)	
-1 to 12 h	16 (42.1)
-12 to 24 h	11 (28.9)
-24 to 36 h	6 (15.8)
-36 to 48 h	5 (13.2)
Comorbidities (%)	
-Diabetes	28 (73.7)
-Obesity	10 (26.3)
-Arteriopathy	9 (23.7)
-Cardiovascular	24 (63.2)
-Neurological	0 (0)
-Chronic kidney disease	2 (5.3)
-Pulmonary	1 (2.6)
-Cirrhosis	3 (7.9)
-Clostridioides difficile infection	2 (5.3)
-COVID-19 pneumonia	1 (2.6)
-Charlson comorbidity index (mean ± stdev)	6.68 ± 1.50
Laboratory: Mean ± SD *	
Median (IQR) **	
-Hemoglobin	10.9 ± 2.4
-White blood cell count	12,190 (8350–18,052)
-Neutrophil count	9450 (5528–15,325)
-NLR	7.48 (4.11–14.68)
-Platelet count	305,414 ± 162,171
-C-Reactive Protein ***	125.4 (89.9–183.13)
-Albumin ***	2.33 ± 0.55
-INR	1.25 (1.10–1.49)
-Urea	51 (32–97)
-Creatinine	0.94 (0.71–1.90)
Imaging diagnosis—CT scan, MRI (%)	3 (7.9)
Isolated flora (%)	
-monomicrobial	20 (52.6)
-polymicrobial	11 (28.9)
-not done/sterile	6/1 (15.8/2.6)
Blood cultures	
-Positive/negative (%)	5/3 (62.5)
-Species	
Complications (%)	
-Septic shock	12 (31.6)
-Acute kidney injury	10 (26.3)
Antibiotherapy (%)	
-Adequate	21 (55.3)
-Inadequate	8 (21.1)
-Not assessed	9 (23.7)
Therapy (%)	
-Surgery	38 (100)
-Amputation	10 (26.3)
-Reintervention	11 (28.9)
-Vacuum	12 (31.6)
-Hospitalisation stay (days, median)	12.5
-In-hospital mortality (%)	11 (28.9)

NLR = Neutrophil-to-lymphocyte Ratio. * Normal distribution was noted for hemoglobin, platelet count, albumin, and CCI. ** White blood cell and neutrophil counts, NLR, INR, C-reactive protein, urea, and creatinine had a non-normal distribution. *** For C-reactive protein and albumin values, mean and standard deviations were estimated in only 13 cases.

**Table 2:** Isolated strains in necrotizing fasciitis.

	All No (%)	Upper Limb No (%)	Lower Limb No (%)
*Staphylococcus aureus*	13 (41.9)	2 (33.3)	12 (48)
*Streptococcus* spp.	7 (22.6)	1 (16.7)	6 (24)
*Acinetobacter baumanii*	6 (19.4)	1 (16.7)	5 (20)
*E. coli*	6 (19.4)	0 (0)	6 (24)
*Klebsiella* spp.	6 (19.4)	2 (33.3)	4 (16)
*Enterococcus* spp.	3 (9.7)	1 (16.7)	2 (8)
*Pseudomonas aeruginosa*	2 (6.5)	0 (0)	2 (8)
*Proteus mirabilis*	2 (6.5)	0 (0)	2 (8)
*Staphylococcus coagulase-negative*	1 (3.2)	0 (0)	1 (4)
*Enterobacter* spp.	1 (3.2)	0 (0)	1 (4)
*Peptostreptococcus*	1 (3.2)	0 (0)	1 (4)
*Serratia*	1 (3.2)	0 (0)	1 (4)
TOTAL	49	7	43

All percentages are based on the 31 patients with extremity fasciitis and positive cultures (upper limb: *n* = 6; lower limb: *n* = 25).

**Table 3:** Risk factors associated with mortality in necrotizing fasciitis.

	Deaths *n* = 11	Alive *n* = 27	OR (Categorical)	*p*-Value
CATEGORICAL				
Gender M/F (%M)	7/4 (63.6)	22/5 (81.5)	0.326	0.241
Site (lower limb/upper limb)	9/2 (81.8/18.2)	23/4 (85.2/14.8)	0.783	0.797
Comorbidities				
-Diabetes	9 (81.8)	19 (70.4)	0.895	0.472
-Obesity	5 (45.5)	5 (18.5)	3.967	0.097
-Peripheral arterial disease	4 (36.4)	5 (18.5)	2.514	0.248
-Cardiovascular disease	6 (45.4)	18 (66.7)	0.600	0.484
-Cirrhosis	2 (18.2)	1 (9.1)	5.778	0.172
-Pulmonary disease	1 (3.7)	1 (9.1)	2.600	0.514
-Chronic kidney disease	1 (3.7)	1 (9.1)	2.600	0.514
-*Clostridioides difficile* infection	0 (0)	2 (7.4)	0.444	0.610
-COVID-19 pneumonia	1 (3.7)	0 (0)	7.857	0.218
Isolated flora (poly/monomicrobial)	4/5 (36.4/45.5)	7/15 (25.9/55.6)	1.7143	0.507
Flora type (Gram-negative/positive)	8/1 (63.6/9.1)	9/13	11.5556	0.033
Complications				
-Septic shock	7/4 (63.6/36.4)	5/22 (18.5/81.5)	7.700	0.011
-Acute kidney injury	9/2 (81.8/18.2)	1/26 (3.7/96.3)	117.000	0.000
Antibiotherapy (adequate/inadequate)	6/3 (54.6/27.3)	15/5 (55.6/18.5)	0.6667	0.643
Amputation	2 (18.2)	8 (29.6)	0.528	0.472
CONTINUOUS				
Age mean ± stdev (min–max)	57.8 ± 16.3 (29–90)	57 ± 12 (20–76)		0.932
Laboratory: mean ± std dev *				
Median (IQR) **				
-Hemoglobin	9.9 ± 2.2	11.4 ± 2.4		0.077
-White blood cell count	13,000 (6165–29,290)	11,380 (8831–17,685)		0.772
-Neutrophil count	11,000 (4540–27,425)	7900 (6106–15,050)		0.974
-NLR	9.17 (7.3–23.65)	6.92 (3.81–13.27)		0.077
-Platelet count	213,066 ± 162,118	343,038 ± 152,483		0.032
-INR	1.56 (1.42–1.98)	1.18 (1.10–1.42)		0.015
-Albumin	2.29 ± 0.74	2.39 ± 0.29		0.532
-C-Reactive Protein	183.13 (125.4–271)	93.15 (73.19–157.49)		0.164
-Urea	141 (87–182)	43 (23–61)		0.000
-Creatinine	2.21 (1.71–4.24)	0.80 (0.69–1.07)		0.001
-CCI	7.2 ± 1.5	6.3 ± 1.4		0.110

NLR = Neutrophil-to-lymphocyte Ratio. CCI = Charlson comorbidity index. * Normal distribution was noted for hemoglobin, platelet count, albumin, and CCI. ** White blood cell and neutrophil counts, NLR, INR, C-reactive protein, urea, and creatinine had a non-normal distribution.

**Table 4:** Firth penalized regression for in-hospital mortality (univariate and multivariate analysis).

Factor	Univariate Analysis Odds Ratio (95% CI), *p*	Multivariate Analysis Odds Ratio (95% CI, *p*)
Obesity	3.81 (0.84, 21.84), 0.082	4.25 (0.36, 73.12), 0.222
Acute kidney injury	**67.13 (7.07, 1851.52), <0.0001**	**24.31 (1.21, 1554.20), 0.019**
Septic shock	**6.82 (1.46, 40.54), 0.013**	1.34 (0.09, 21.08), 0.807
Gram-negative vs. positive	4.73 (0.97, 30.12), 0.054	4.38 (0.33, 99.41), 0.237
INR (binary, 1.5 cutoff)	**13.91 (1.81, 335.71), 0.0004**	1.14 (0.05, 23.64), 0.923
NLR (binary, 10 cutoff)	**13.31 (1.73, 319.45), 0.0005**	1.48 (0.06, 32.55), 0.778
PLT (binary, 150,000 cutoff)	**5.23 (1.13, 29.58), 0.024**	1.12 (0.08, 15.34), 0.927
Hb value (binary, 10 g/dL cutoff)	3.51 (0.03, 11.23), 0.104	1.30 (0.08, 17.65), 0.835

Values with statistical significance in univariate and multivariate analysis were marked with bold font.

**Table 5:** Risk factors associated with amputation in necrotizing fasciitis.

	Amputation (*n* = 10)	No Amputation (*n* = 28)	OR (Categorical)	*p*-Value
CATEGORICAL				
Gender M/F (%)	10/0 (100/0)	19/9 (67.9/32.1)	10.231	0.121
Lower limb/upper limb	10/0 (100/0)	22/6 (78.6/21.4)	6.067	0.234
Comorbidities				
-Diabetes	9 (90)	19 (67.9)	4.263	0.199
-Obesity	1 (10)	10 (35.7)	0.2	0.153
-Peripheral arterial disease	3 (30)	6 (21.4)	1.571	0.586
-Cardiovascular	7 (70)	16 (57.1)	1.750	0.478
-Respiratory	1 (10)	1 (3.6)	3.000	0.4535
-Renal	2 (20)	0 (0)	16.765	0.077
-Cirrhosis	0 (0)	3 (10.7)	0.347	0.496
-Clostridioides difficile infection	0 (0)	2 (7.1)	0.505	0.667
-COVID-19	0 (0)	1 (3.6)	0.873	0.935
Isolated flora (poly/monomicrobial)	5/3 (50/30)	15/8 (53.6/28.6)	0.889	0.890
Gram-negative vs. positive	4/4 (40/40)	13/10 (46.4/35.7)	0.769	0.7498
Complications				
-Septic shock	3 (30)	9 (32.1)	0.900	0.9048
-Acute kidney injury	3 (30)	7 (25)	1.2857	0.758
Antibiotherapy A/I/ND (%)	3/4/3(30/40/30)	17/5/6 (60.7/17.8/21.5)	0.226	0.100
Death	2 (20)	9 (32.1)	0.528	0.472
Vacuum	3 (30)	9 (32.1)	0.9048	0.900
CONTINUOUS				
Age mean ± std. dev. (min–max)	55.2 ± 12.8 (29–72)	58.4 ± 13.0 (20–90)		0.512
Laboratory: Mean ± std *				
Median (IQR) **				
-Hemoglobin	11.4 ± 2.3	9.9 ± 2.2		0.081
-White blood cell count	15,800 (11,650–17,595)	9500 (7577–18,402)		0.345
-Neutrophil count	11,750 (8675–14,625)	7007 (4884–15,282)		0.312
-NLR	6.53 (4.45–8.93)	7.71 (4.51–17.65)		0.456
-Platelet count	350,420 ± 127,425	289,341 ± 170,034		0.308
-INR	1.35 (1.19–1.49)	1.24 (1.09–1.51)		0.436
-Albumin	2.08 ± 0.44	2.41 ± 0.56		0.294
-C-Reactive Protein	92.9 (70.4–96)	182.7 (125.4–271)		0.076
-Urea	62 (26–118)	51 (35–92)		1.000
-Creatinine	1.41 (0.90–2.18)	0.82 (0.71–1.80)		0.362

* Normal distribution was noted for hemoglobin, platelet count, and albumin. ** White blood cell and neutrophil counts, NLR, INR, C-reactive protein, urea, and creatinine had a non-normal distribution.

## Data Availability

The datasets obtained and used during the current study are available upon request from the corresponding authors.

## Supplementary Materials

The following supporting information can be downloaded at https://www.mdpi.com/article/10.3390/medicina62091744/s1.
